# Impact of Nonresonant Intense Laser and Electric Fields on a Low-Dimensional CdTe/CdSe Type-II Cone

**DOI:** 10.3390/nano15151208

**Published:** 2025-08-07

**Authors:** Fredy Amador Donado, Fernando Guerrero Almanza, Camilo Frías Viña, Juan Alejandro Vinasco, J. Sierra-Ortega, Gene Elizabeth Escorcia-Salas, R. V. H. Hahn, M. E. Mora-Ramos, O. Mommadi, A. El Moussaouy, R. Boussetta, D. Duque, A. L. Morales, S. Uran-Parra, C. A. Duque

**Affiliations:** 1 Grupo de Investigación en Teoría de la Materia Condensada, Universidad del Magdalena, Santa Marta 470004, Colombia; fredyamador@unicesar.edu.co (F.A.D.); fernandoguerreroja@unimagdalena.edu.co (F.G.A.); camilofriasav@unimagdalena.edu.co (C.F.V.); jcsierra@unimagdalena.edu.co (J.S.-O.); geneescorcias@unicesar.edu.co (G.E.E.-S.); 2 Grupo de Óptica e Informática, Departamento de Física, Universidad Popular del Cesar, Sede Hurtado, Valledupar 200001, Colombia; 3 Departamento de Ciencias Básicas, Facultad de Ingeniería y Administración, Universidad Nacional de Colombia Sede Palmira, Palmira 763531, Colombia; juan.vinascos@udea.edu.co; 4 Departamento de Electrónica y Tecnología de Computadores, Facultad de Ciencias, Universidad de Granada, 18071 Granada, Spain; hahn@ugr.es; 5 Centro de Investigación en Ciencias-IICBA, Universidad Autónoma del Estado de Morelos, Av. Universidad 1001, Cuernavaca 62209, Morelos, Mexico; memora@uaem.mx; 6 Research Laboratory in Sciences and Techniques, ESEFA, Ibnou Zohr University, Agadir 80000, Morocco; omommadi@gmail.com; 7 Laboratory of Innovation in Science, Technology and Education, CRMEF, Oujda 60000, Morocco; azize10@yahoo.fr; 8 Faculty of Science and Technology, Department of Physics, Abdelmalek Essaâdi University, Al Hoceima 32003, Morocco; boussettareda7@gmail.com; 9 GICEI, Facultad de Ingeniería, Institución Universitaria Pascual Bravo, Medellín 050034, Colombia; duque.derfrey@gmail.com; 10 Grupo de Materia Condensada-UdeA, Instituto de Física, Facultad de Ciencias Exactas y Naturales, Universidad de Antioquia UdeA, Calle 70 No. 52-21, Medellín 050010, Colombia; alvaro.morales@udea.edu.co (A.L.M.); salomon.uran@udea.edu.co (S.U.-P.)

**Keywords:** truncated conical quantum dot, electron states, hole states, exciton states, nonresonant intense laser field, electric field

## Abstract

In this work, a theoretical study on the combined effects of an external electric field and a nonresonant intense laser field on the electronic properties of a quantum dot with a truncated cone shape is presented. This quantum dot was made from a type-II CdTe/CdSe heterostructure (core/shell). Using the effective mass approximation with parabolic bands and the finite element method, the Schrödinger equation was solved to analyze the confined states of electron, hole, and exciton. This study demonstrates the potential of combining nonresonant intense laser and electric fields to control confinement properties in semiconductor nanodevices, with potential applications in optoelectronics and quantum mechanics-related technologies.

## 1. Introduction

Over the last few decades, semiconductor quantum dots (QDs) have emerged as a powerful class of low-dimensional systems, owing to their size-tunable quantum confinement effects and the potential for integration into various optoelectronic devices [1,2,3]. Among the diverse geometries explored, epitaxially grown QDs with nontrivial morphologies, such as truncated conical or lens-shaped structures, have garnered significant interest for their enhanced control over carrier localization and optical anisotropy. These geometries are typically realized using Stranski–Krastanov (SK) growth modes in epitaxial systems, such as GaAs/AlGaAs or CdTe/CdSe, where strain-driven self-assembly enables the formation of well-defined conical nanostructures [4,5,6]. The truncated conical geometry is representative of epitaxially grown nanostructures obtained via self-assembly techniques such as SK growth. These nanostructures typically form during lattice-mismatched deposition processes on substrates and have been reported to exhibit various morphologies, including pyramidal, dome-shaped, and truncated cone shapes. In particular, the formation of CdTe/CdSe core/shell type-II QDs with epitaxial shells has been experimentally demonstrated in noncoordinating solvents, where shell growth proceeds with high crystallinity and quantum efficiency enhancement [7].

The unique electronic properties of QDs make them ideal candidates for a wide variety of applications in fields such as medicine [8,9], quantum computation [10], solar cells [11,12], catalysis [13,14], photodetectors [15], and optoelectronics [16,17,18,19,20,21]. In particular, QDs have gained increasing attention in micro-LED displays, where their tunable emission and high color purity make them ideal for color conversion and integration with inorganic LEDs [20,21]. Concerning the latter, QL-based light-emitting diodes deserve special mention, given their impact on daily life since their implementation in television screens, which improves color representation and significantly enhances the viewing experience [22,23,24]. It is also worth mentioning that control over size and morphology has allowed us to develop synthesis protocols for nanoparticles with nontrivial geometries [25,26,27,28].

In particular, branched nanocrystals such as tetrapods [29,30], single and double quantum rings [31,32,33], and conical QDs [34,35,36] have emerged as strong alternatives for numerous applications due to the peculiar optoelectronic and mechanical properties they exhibit as a result of their nontrivial geometry. For example, engineering their size and composition leads to specific optical properties, such as narrow emission line widths or high photoluminescence quantum yields, which are crucial to designing high-performance photonic devices, including lasers, displays, and sensors. The case of conical QDs is significant due to the possibility of transitioning from a quantum wire for a strongly prolate cone to a quantum well in the case of a strongly oblate one [34]. In addition, by adjusting the geometric parameters of GaAs conical QDs, an ensemble can be modeled to prepare RGB-LED devices, provided that the interband transition energy falls within the visible part of the spectrum [37]. Theoretical studies have also demonstrated that for conically shaped QDs, the absorption peak can be shifted to lower incident light energies by externally applying an electric field with increasing intensity [34], while a decrease in size results in a blueshift of the absorption peak of GaAs conical QDs [35].

Compared with well-established nanostructures such as quantum wells, wires, and rings, truncated conical quantum dots (TCQDs) exhibit a distinct combination of geometric asymmetry and multi-dimensional confinement, making them especially versatile for tunable optoelectronic applications. Quantum wells provide strong vertical confinement but lack flexibility in radial control [38,39,40]. At the same time, quantum rings are excellent for exploring Aharonov–Bohm-like phenomena, yet demand near-ideal symmetry and often offer limited control over longitudinal confinement [31,32,33]. Quantum wires, such as CdSe/CdTe heterostructure nanowires under uniaxial strain, have been shown to exhibit tunable band offsets and confinement properties [41]. However, the overlap between electron and hole states is generally low, and their behavior is dominated by 1D confinement and axial strain. Similarly, multilayer cylindrical wires under pressure and strain can control exciton properties such as radiative lifetime and interband transition energy [42], but still lack the geometric asymmetry that allows spatial wavefunction engineering in multiple directions. In contrast, truncated conical QDs offer a hybrid regime between QDs and wires, enabling smooth tuning from strong to weak confinement regimes via base radius, height, and shell thickness. The vertical asymmetry inherent in their shape makes them more sensitive to electric fields and laser-induced dressing potentials, allowing localized confinement control that is challenging to achieve in flat or cylindrical geometries [34,35,36,37]. Moreover, the ability to induce transitions from ring-like to dot-like behavior under field modulation (as will be shown in this work) gives TCQDs a unique advantage for light-emitting devices, where spatial overlap and confinement tuning directly impact emission efficiency.

Advances in chemical synthesis techniques have made it possible to fabricate nanoparticles (NPs) composed of two or more different crystal phases, e.g., core/shell and multilayered QDs, which combine several components with different electronic structures in a single entity. The precise band engineering at the interface of these heterostructures plays an essential role in the realization of new functional optoelectronic devices, thanks to the formation of type-I and type-II heterostructures [43,44]. Type-II band alignments, especially in CdTe/CdSe core/shell NPs, are ideal candidates for their application in photovoltaics, given their long-range photoinduced charge separation [44,45,46].

The interaction of QDs with a nonresonant intense laser field (ILF) and electric or magnetic fields represents an exciting field of research. It offers opportunities to understand and design advanced materials and devices with specific properties. Combining these factors in the study of TCQDs within type-II heterojunctions adds complexity and potential for future innovative technological applications. In this study, we delve into a detailed exploration of type-II semiconductor nanostructures with a truncated conical geometry. The electron, hole, and exciton states are obtained for a CdTe/CdSe core/shell TCQD with fixed size parameters. They are subjected to nonresonant intense radiation, polarized along the *z*-axis, and the influence of an external electric field applied along the *z*-direction. In this way, we demonstrate that it is possible to model irregularities or changes in the geometry of real systems prior to their experimental fabrication.

This paper is organized as follows: Section 2 presents the theoretical model and describes the underlying theory. Section 3 presents and discusses our numerical results, and Section 4 provides our main conclusions.

## 2. Theoretical Framework

**Figure 1:**
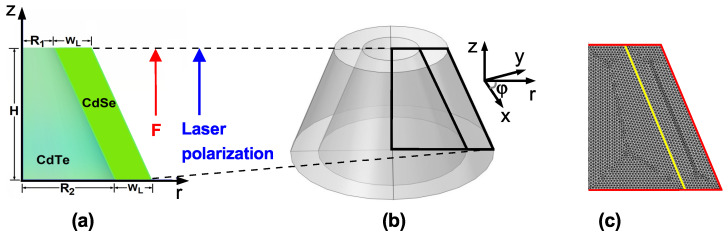
Schematic representation of the (**a**) cross-section and (**b**) 3D view of the core/shell CdTe/CdSe TCQD with fixed geometrical parameters $R_{1}=3\;$nm, $R_{2}=8\;$nm, $W_{L}=3\;$nm, and H=12nm. The directions of both the electric field and the polarization of the laser field are also shown. The core/shell materials are CdTe/CdSe. A vacuum surrounds the heterostructure. In (**c**) the rz-plane of the structure is depicted, where the mesh for the finite element method calculation is shown. Highlighted lines in (**c**) indicate the main boundaries of the problem.

This study focuses on the electron, hole, and exciton energy states and their associated wavefunctions in a CdTe/CdSe core/shell TCQD with fixed size parameters under an external electric and/or nonresonant ILF. To gain insights into the problem, a schematic representation of the core/shell TCQD geometry is provided in Figure 1, along with the main dimensions of the nanostructure and the directions of the electric and laser fields. For clarity, the reference system is also depicted, using Cartesian (x,y,z) and cylindrical coordinates (r,φ,z). A cross-section of the structure (with φ=0) is shown in Figure 1a, where the two materials that make up the TCQD can be easily identified, i.e., CdSe and CdTe. The latter constitutes the $D_{1}$-domain and displays a barrier-like behavior, while the former presents a well-like behavior and constitutes the $D_{2}$-domain. This configuration is advantageous for building a 2D axisymmetric model where the azimuthal symmetry simplifies the numerical solution of the Schrödinger equation. Figure 1b shows a 3D view of the TCQD obtained by rotating around the *z*-axis in Figure 1a. For calculations, the origin of the reference system is set at the center of the TCQD base. Finally, in Figure 1c, the rz-plane of the structure is depicted, where the mesh for the FEM calculation is shown.

The transformation of the system under a nonresonant laser field can be modeled using a *z*-linearly polarized monochromatic field which is incident, for example, along the *x*- or *y*-transversal directions; the vector potential takes the form

(1) $$A\left(t\right)=A_{0}\cos\left(\omega t\right)\;\hat{z}\;,$$

where $A_{0}$ and ω are the amplitude and frequency of the laser field and $\hat{z}$ denotes the unit vector along the *z*-direction.

The time-dependent Hamiltonian of the problem, within the effective mass approximation, is written in cylindrical coordinates for electrons and holes as follows:

(2) $$\frac{{\left[\hat{p}-q\;A\left(t\right)\right]}^{2}}{2\;m_{c,l}^{*}\left(r,z\right)}\psi \left(r,\varphi ,z\right)+\left[V(r,z)-qFz\right]\psi \left(r,\varphi ,z\right)=-\frac{\hbar }{i}\frac{\partial }{\partial t}\psi \left(r,\varphi ,z\right)\;,$$

where $\hat{p}=-i\;\hbar \;\nabla$, $m_{c,l}^{*}$ is the effective mass in the inner material (CdTe, l=1) or the outer material (CdSe, l=2); c=e,h for the electron and hole, respectively; and $q=-q_{e}$ for the electron and $q=+q_{e}$ for the hole with $q_{e}$ being the absolute value of the elemental charge. Additionally, *F* designates the electric field magnitude, and *V* is the potential value in each of the two materials; for electrons, V=0 for CdSe, and V=0.420 eV in CdTe. In contrast, for the hole, V=0 in CdTe, and V=0.57 eV in CdSe.

Equation (2) can be simplified using the Kramers–Henneberger transformation [47,48]. This technique incorporates the effect of the laser field by transferring the time dependence from the kinetic term to the potential term [49]. For this reason, the transformation is widely referred to as the laser dressing of the potential. With this, the time-dependent Schrödinger equation takes the time-independent form given by

(3) $$-\frac{\hbar^{2}}{2\;m_{c,l}^{*}}\;\nabla^{2}\;\psi \left(r,\varphi ,z\right)+\left[V_{d}\left(r,z,\alpha_{0}\right)-q\;F\;z\right]\;\psi \left(r,\varphi ,z\right)=E\;\psi \left(r,\varphi ,z\right)\;,$$

with a laser parameter for electrons,

(4) $$\alpha_{0}\left(r,z\right)=\left\{\begin{array}{cc} \alpha_{0}, & \mathrm{if}\;\left(r,z\right)\in D_{2}\\ \alpha_{0}\;\frac{m_{e,2}^{*}}{m_{e,1}^{*}}, & \mathrm{if}\;\left(r,z\right)\in D_{1}, \end{array}\right.$$

and one for the hole,

(5) $$\alpha_{0}^{\prime }\left(r,z\right)=\left\{\begin{array}{cc} \alpha_{0}\;\frac{m_{e,2}^{*}}{m_{h,1}^{*}}, & \mathrm{if}\;\left(r,z\right)\in D_{2}\\ \alpha_{0}\;\frac{m_{e,2}^{*}}{m_{h,2}^{*}}, & \mathrm{if}\;\left(r,z\right)\in D_{1}\;. \end{array}\right.$$

The transformation corresponds to a change from the laboratory frame to a non-inertial frame that oscillates with the classical motion of an electron under a laser field. This approach is valid in the high-frequency regime, where the laser period is much shorter than the characteristic timescales of the system. In this framework, the electron experiences a cycle-averaged potential, also known as laser-dressed potential, which captures the net effect of the oscillating field. The transformed effective potential is given by

(6) $$V_{d}\left(r,z,\alpha_{0}\right)=\frac{\omega }{2\pi }\int_{0}^{\frac{2\pi }{\omega }}V\left(r,z+\alpha_{0}\left(r,z\right)\cos\omega t\right)\;dt,$$

where $\alpha_{0}$ is the classical displacement of the particle under the oscillating laser field. This displacement is defined as $\alpha =\frac{e\;A_{0}}{m_{c,l}^{*}\;\omega^{2}}$, with $A_{0}$ representing the amplitude of the classical oscillation induced by the laser field and connected directly to the laser intensity. These expressions justify the use of the time-averaged potential, which effectively describes the modified confinement landscape because of the presence of the high-frequency external field.

Physically, the laser-dressed potential $V_{d}\left(r,z,\alpha_{0}\right)$ in Equation (6) can be interpreted as the time-averaged potential experienced by the electron in the Kramers–Henneberger frame, where the effect of the laser field is incorporated into a modified spatial profile of the confinement potential. This approach captures the influence of the electron’s quiver motion under a high-frequency nonresonant laser field, effectively reshaping the potential landscape without requiring explicit time evolution of the wavefunction. As a result, one obtains a time-independent Schrödinger equation that is computationally more tractable while still retaining the key physical effects of the laser field.

The solution of Equation (3) was sought under the assumption of parabolic bands using the finite element method (FEM) as implemented in COMSOL-Multiphysics software (6.1 version) [50,51,52]. The boundary conditions were imposed in the following way: (i) wavefunction continuity at the core/shell interface: $\Psi_{core}=\Psi_{shell}$; (ii) BenDaniel–Duke boundary condition (yellow contour in Figure 1c): $\frac{1}{m_{core}^{*}}\frac{\partial \Psi_{core}}{\partial X}=\frac{1}{m_{shell}^{*}}\frac{\partial \Psi_{shell}}{\partial X}$ with X=r,z; and (iii) Dirichlet boundary conditions on the outer surface of the CdTe/CdSe core/shell TCQD (red contour in Figure 1c): Ψ=0. For computational efficiency, the system is divided into two regions as indicated in Figure 1a: the first region consists of CdTe, while the second consists of CdSe.

By expanding the first term in Equation (3) and applying the azimuthal symmetry condition of the system, it is possible to assume a solution in cylindrical coordinates of the form ψ(x,y,z)=ψ(ρ,φ,z)=R(ρ,z)$e^{\;i\;n\;\varphi }$. As a consequence, the function R(ρ,z) must satisfy the following differential equation:

(7) $$\left[-\frac{\hbar^{2}}{2\;m_{c,l}^{*}}\;\nabla^{2}\;+\frac{\hbar^{2}\;n^{2}}{2m_{c,l}^{*}\;r^{2}}+\left[V_{d}\left(r,z,\alpha_{0}\right)-q\;F\;z\right]\right]\;R\left(r,z\right)=E\;R\left(r,z\right)\;,$$

where n∈Z is the azimuthal quantum number and $\nabla^{2}$ is the two-dimensional Laplacian operator in the polar coordinates.

During the calculations, Equations (3) and (7) are used as the two fundamental models to be solved. Equation (3) provides a general framework for describing the behavior of the system under the influence of external fields. In particular, Equation (7) reveals the quantum number associated with each state, which enables the identification of the type of wavefunction involved, thus offering a more detailed characterization of the electronic states. Moreover, since Equation (7) corresponds to a 2D axisymmetric model, it significantly reduces the computational cost compared with a complete 3D calculation while still capturing the essential physics of the system.

After obtaining the uncorrelated ground-state wavefunctions for the electron and hole, denoted by $\psi_{e}^{1}\left({\vec{r}}_{e}\right)$ and $\psi_{h}^{1}\left({\vec{r}}_{h}\right)$, respectively, we can evaluate the excitonic contribution from the Coulomb interaction between the two charges. Within the framework of first-order perturbation theory, the Coulomb integral takes the form

(8) $$E_{eh}=\frac{q^{2}}{4\;\pi \;\epsilon_{0}\;\epsilon_{r}}\int_{\Omega_{h}}\int_{\Omega_{e}}\frac{{\left|\psi_{e}^{1}\left({\vec{r}}_{e}\right)\right|}^{2}{\left|\psi_{h}^{1}\left({\vec{r}}_{h}\right)\right|}^{2}}{\left|{\vec{r}}_{e}-{\vec{r}}_{h}\right|}dV_{e}\;dV_{h},$$

where $dV_{e}=\rho_{e}\;d\rho_{e}\;dz_{e}\;d\varphi_{e}$ and $dV_{h}=\rho_{h}\;d\rho_{h}\;dz_{h}\;d\varphi_{h}$ are the volume differentials in cylindrical coordinates for the electron and hole, respectively. The integration domains $\Omega_{h}$ and $\Omega_{e}$ indicate the full volume of the cone represented in Figure 1b.

The angular dependence in Equation (7) can be integrated analytically because of the azimuthal symmetry. The six-dimensional integral becomes four-dimensional. The Coulomb term then simplifies to

(9) $$E_{eh}=\frac{q^{2}}{4\;\pi \;\varepsilon_{0}\;\varepsilon_{r}}\int_{S_{h}}\int_{S_{e}}{\left|\Psi_{e}^{1}\left(\rho_{e}\;,z_{e}\right)\right|}^{2}{\left|\Psi_{h}^{1}\left(\rho_{h}\;,z_{h}\right)\right|}^{2}\;\left[\frac{8\pi \;K\left(\frac{r_{p}}{1+r_{p}}\right)}{r\;\sqrt{1+r_{p}}}\right]\;dV_{e}^{\prime }\;dV_{h}^{\prime }\;,$$

where $r_{p}=\frac{4\rho_{e}\;\rho_{h}}{r^{2}}$, $r=\sqrt{{\left(\rho_{e}-\rho_{h}\right)}^{2}+{\left(z_{e}-z_{h}\right)}^{2}}$, K(x) is a complete elliptic integral of the first kind, $dV_{e}^{\prime }=\rho_{e}\;d\rho_{e}\;dz_{e}$, and $dV_{h}^{\prime }=\rho_{h}\;d\rho_{h}\;dz_{h}$. Here, the domain region for the integrals corresponds to the surface φ=0 depicted in Figure 1a.

## 3. Results and Discussion

The Schrödinger equation, given in Equation (3), was solved using the FEM via COMSOL-Multiphysics software (6.1 version) [50,51,52] within the effective mass approximation. The mesh for the FEM calculations is illustrated in Figure 1c. It was refined locally in the regions where the electron or hole wavefunctions were expected to be localized, to improve accuracy in solving the Schrödinger equation. For the mesh, the following parameters were used: number of vertices, 6862; number of elements, 13,448; and triangular mesh type. These refinements and conditions were carefully selected to strike a balance between numerical precision and computational efficiency.

We investigated numerically the lowest confined energy states for electrons and holes in a CdTe/CdSe core/shell TCQD subjected to an electric and/or nonresonant intense laser field as a function of the intensity of the electric field, *F*, and the laser field parameter, $\alpha_{0}$. The parameters for electrons and holes adopted in the numerical calculations are $m_{e,1}^{*}=0.096\;m_{0}$, $m_{e,2}^{*}=0.120\;m_{0}$, $m_{h,1}^{*}=0.40\;m_{0}$, and $m_{h,2}^{*}=0.45\;m_{0}$ (here, $m_{0}$ is the mass of free electrons). The barrier heights are $V_{1}=420$ meV and $V_{2}=0$ for electrons and $V_{1}=0$ and $V_{2}=0.57$ eV for holes in the CdTe/CdSe TCQD [45]. A relative permittivity of $\varepsilon_{r}=10.2$ was used as a simple average between CdTe and CdSe [53].

**Figure 2:**
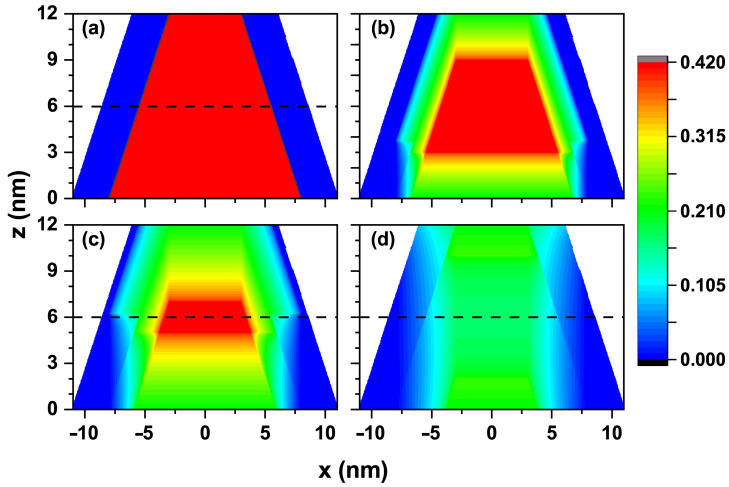
Cross-section of the electron confinement potential in a CdTe/CdSe core/shell TCQD associated with (**a**) $\alpha_{0}=0$, (**b**) $\alpha_{0}=3$ nm, (**c**) $\alpha_{0}=5$ nm, and (**d**) $\alpha_{0}=10$ nm. The geometrical parameters of the structure are kept constant with values $R_{1}=3$ nm, $R_{2}=8$ nm, $W_{L}=3$ nm, and H=12 nm. Additionally, F=0.

**Figure 3:**
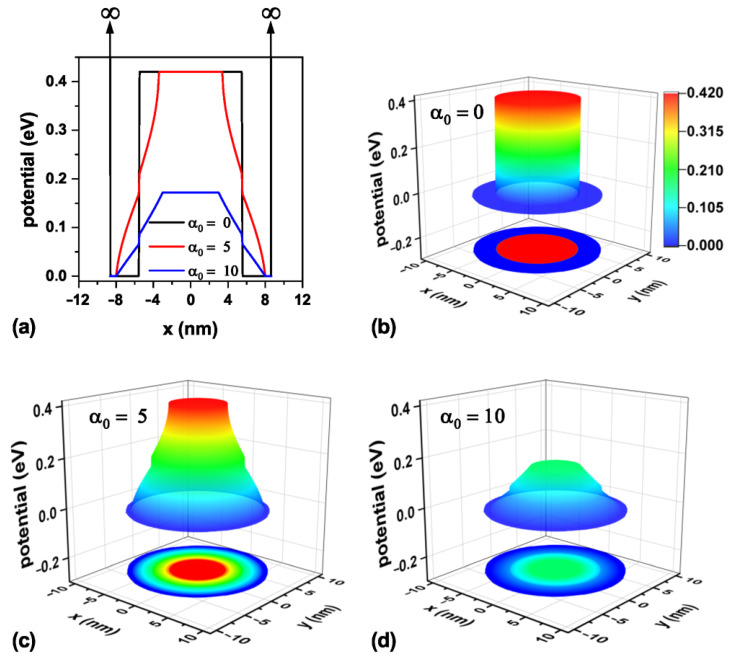
Representation of the electron confinement potential on a transverse section at half the nanostructure height, z=6 nm, for the CdTe/CdSe core/shell TCQD with geometrical parameters H=12 nm, $R_{1}=3$ nm, $R_{2}=8$ nm, and $W_{L}=3$ nm considering three different values of the laser parameter: (**a**) a 2D plot of the potential profile; 3D views and 2D projections of the confinement potential (**b**) in the absence of ILF ($\alpha_{0}=0$), (**c**) with $\alpha_{0}=5$ nm, and (**d**) with $\alpha_{0}=10$ nm. Additionally, F=0.

Figure 2 shows a cross-sectional profile of the confinement potential for electrons in the TCQD. The first panel of this figure corresponds to the absence of a laser field, which allows us to differentiate the boundary between the two building materials, CdSe in blue and CdTe in red. The color scale depicts a range from zero (blue) to the maximum value of 0.420 eV (red), matching the potential values aforementioned for electrons $V_{1}$ and $V_{2}$. The maximum potential at the CdTe core and the rotational symmetry give rise to a wavefunction similar to that obtained in a quantum ring. Figure 2b illustrates how the barrier height and the well width decrease when an ILF of $\alpha_{0}=3$ nm is applied to the nanostructure. At this laser intensity, the potential barrier still creates significant confinement on the electron, preventing its wavefunction from penetrating the core region of the truncated cone. In Figure 2c, the changes in the potential shape resulting from the effect of the external laser field of $\alpha_{0}=5$ nm are exhibited. The red region is remarkably reduced, replaced by intermediate confinement zones, identified by green and light blue colors, making it easier for the electron wavefunction to enter the CdTe core. However, it will not extend to the entire core, as the center still has a barrier. At the base of the cone, the laser causes an irregular confinement region, as the zero potential extends into CdTe. At this laser intensity, the electron exhibits a smaller barrier effect and is more likely to be found at the base, where a wider region of zero potential exists. The effect of the highest intensity laser used in this work, i.e., $\alpha_{0}=10$ nm, is shown in Figure 2d. It is straightforward that the 0.420 eV barrier is completely lost, being now lower in height and, therefore, allowing the wavefunctions of the low-lying electron states to enter the core. The ring behavior exhibited as a result of natural confinement in the type-II heterojunction in the previous cases is largely lost. Finally, the dashed lines plotted at z=6 nm in Figure 2a,c,d correspond to the location of the cut plane in Figure 3 (see below).

The confinement potential associated with the three values of laser parameters analyzed in this work is presented in Figure 3 on a transverse section at half the TCQD height, i.e., z=6 nm. It is important to note that there exists an infinite barrier beginning at r=9 nm and continuing along the cone generatrix, which is independent of the inclusion of the ILF and which, in an actual situation, would represent the air exposure of the outer surface. For clarity, vertical arrows indicating the existence of this infinite barrier are displayed in Figure 3a, which is valid throughout the study. The theoretical model was implemented using the Dirichlet boundary condition. Looking at Figure 3b–d, it is straightforward that an increase in laser field intensity turns into a reduction in the confinement potential barrier for electrons, which will allow for an increasing penetration of the low-lying electron wavefunctions in the core region, which otherwise would not be possible. These effects, modeled by a dressed potential, are equivalent to having an irregular cone, so it would be likely to model irregularities or changes in the geometry of real systems before their experimental fabrication.

**Figure 4:**
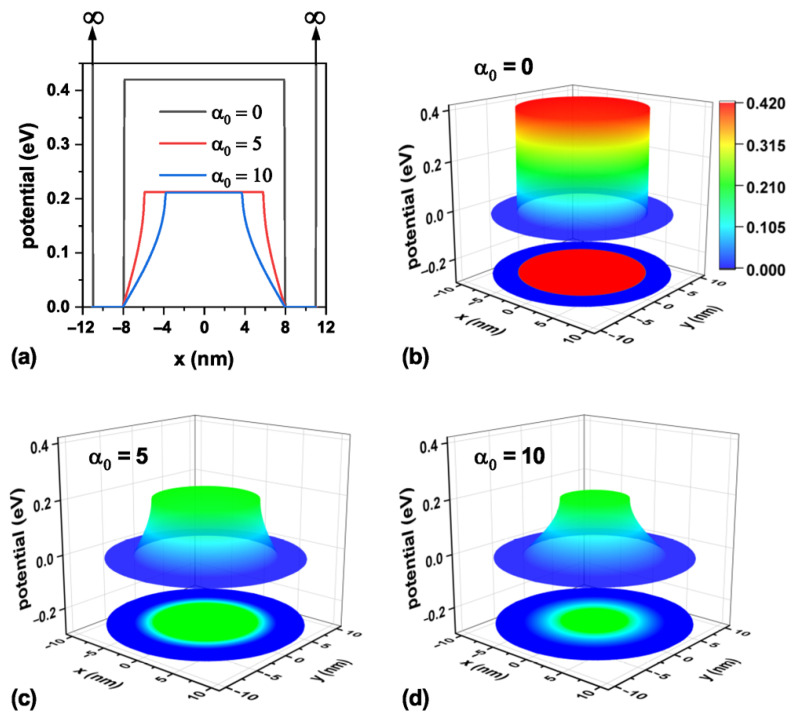
Representation of the electron confinement potential on a transverse section at the base of the nanostructure, z=0 nm, for the CdTe/CdSe core/shell TCQD with geometrical parameters H=12 nm, $R_{1}=3$ nm, $R_{2}=8$ nm, and $W_{L}=3$ nm considering three different values of the laser parameter: (**a**) a 2D plot of the potential profile; 3D views and 2D projections of the confinement potential (**b**) in the absence of ILF, (**c**) with $\alpha_{0}=5$ nm, and (**d**) with $\alpha_{0}=10$ nm. Additionally, F=0.

Analogously, Figure 4 shows the confinement potential associated with the three laser parameter values under study on a transverse section at the TCQD base, i.e., z=0 nm. Following the arguments presented when discussing Figure 3, in the case of Figure 4a, vertical arrows are displayed to indicate the infinite barrier corresponding to the outer surface of the nanostructure, now located at x=±11 nm. From this panel, it is evident that the ILF reduces the barrier both in height and width, resulting in lighter electron confinement. As the laser parameter increases, as shown in Figure 4b–d, the probability of finding the electron in the bottom region of the nanostructure increases due to the diminishing barrier observed in this area. This fact is also reinforced by the results already presented in Figure 3.

**Figure 5:**
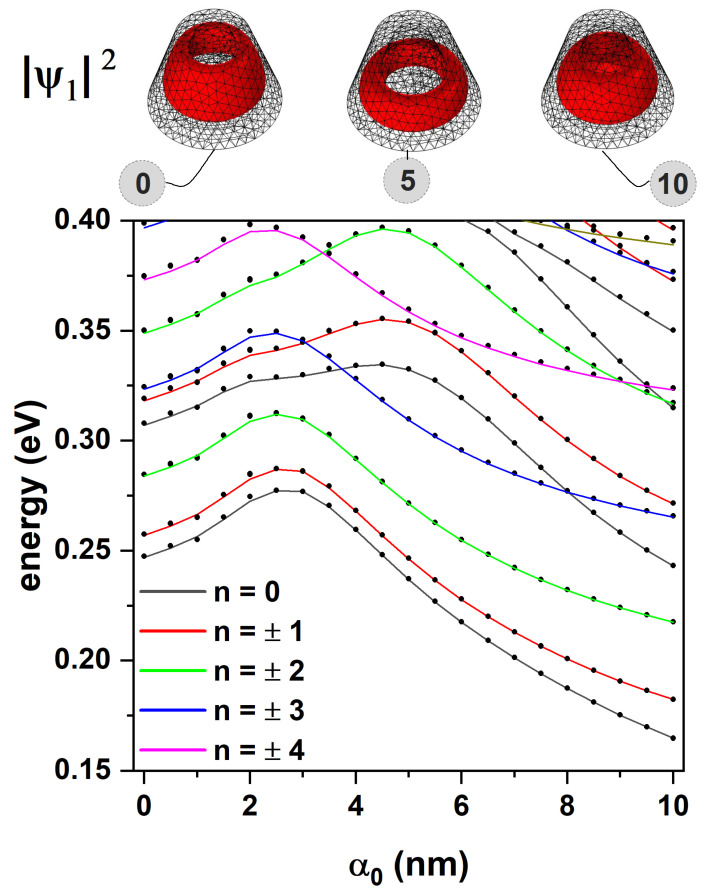
Energy spectrum of the CdTe/CdSe core/shell TCQD with geometrical parameters H=12 nm, $R_{1}=3$ nm, $R_{2}=8$ nm, and $W_{L}=3$ nm as a function of the laser parameter for different values of the quantum number, *n*. Solid lines correspond to the axisymmetric 2D model and dotted lines are obtained from a 3D model. The ground-state electron probability density for three values of the laser parameter is also depicted. The calculations correspond to F=0.

In Figure 5, the energies of an electron confined in the CdTe/CdSe core/shell TCQD presented in Figure 1 are presented as functions of the laser parameter, $\alpha_{0}$. Two sets of results are shown: solid lines are associated with the 2D axisymmetric model, while dotted lines correspond to the 3D model. One of the advantages of using the 2D axisymmetric model is the possibility of unequivocally identifying the quantum number *n*. Additionally, a significant reduction in computational cost is achieved for the 3D model due to its axial symmetry. The potential barrier generated by the different materials is 0.420 eV. It can be seen that the energy of the low-lying states increases with the laser parameter, up to approximately $\alpha_{0}=3$ nm. This is explained by the fact that the width of the well is being reduced while the barrier is still high, resulting in greater electron confinement. From this value on, a gradual decrease in the energies mentioned above is observed, as the well becomes narrower in most of the structure; however, the barrier has dropped sufficiently to decrease its confining effect. Furthermore, as discussed in Figure 3, for values of z<6 nm, the well evolves into a wider shape, which keeps the electron mainly in the bottom region of the TCQD. The combined effect of a lower barrier and a wider well at the base implies less confinement, which is reflected in the drop in energies. For completeness, the electron probability densities associated with the laser parameters $\alpha_{0}=0$, $\alpha_{0}=5$, and $\alpha_{0}=10$ nm are shown at the top of Figure 5 to clarify the effect of the ILF on the nanostructure. When F=0, a quantum ring-like behavior is observed for the electron located within the CdSe region. For an intermediate value of the laser parameter, a more defined quantum ring-like probability density is observed, i.e., a washer-shaped distribution [54]. For the third case, $\alpha_{0}=10$ nm, we have a system that behaves fundamentally as a QD, losing the double connectedness typical of a quantum ring, as pointed out when discussing Figure 2.

The evolution of electron energy, presented in Figure 5, can be understood in terms of how the laser field parameter $\alpha_{0}$ modulates the effective confinement potential. The Kramers–Henneberger transformation used in our model introduces a time-averaged shift in the potential landscape due to the ILF, effectively smoothing and flattening the confinement barrier. For electrons, this results in a non-monotonic energy shift: As $\alpha_{0}$ increases from 0 to ∼3 nm, the barrier remains high, but the well narrows, increasing the confinement energy (leading to a rise in energy levels). Beyond this point, however, the barrier itself is substantially reduced (as shown in Figure 2d and Figure 3d), and the electron wavefunctions begin to penetrate deeper into the CdTe core, reducing the overall confinement and causing the energy levels to drop. To illustrate this, we note that for $\alpha_{0}=0$, the effective potential confines the electron entirely within the CdSe shell. As $\alpha_{0}$ increases to 10 nm, the potential in the CdTe core drops by up to ∼60%, dramatically improving the penetration depth of the wavefunction (see insets of Figure 5). This transition causes the system to evolve from a ring-like (doubly connected) to a dot-like (singly connected) regime. The energy minimum observed around $\alpha_{0}\approx$ 3–4 nm represents the point of maximum confinement before delocalization begins due to potential flattening.

**Figure 6:**
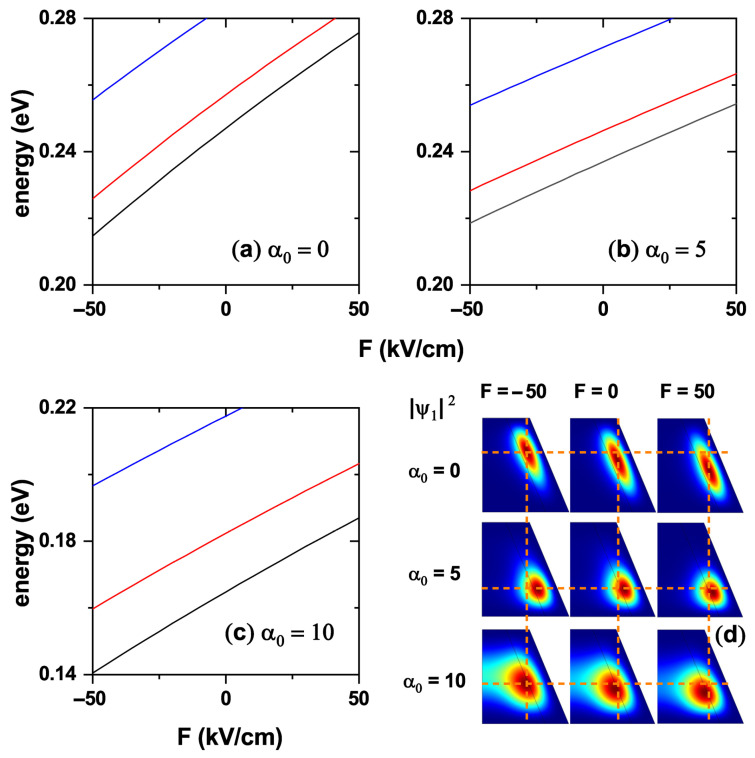
Energies of the three low-lying electron states in the CdTe/CdSe core/shell TCQD are presented as functions of the externally applied electric field for three different values of the laser parameter: (**a**) $\alpha_{0}=0$, (**b**) $\alpha_{0}=5$, and (**c**) $\alpha_{0}=10$ nm. In (**d**), the electron ground-state probability densities are shown for three different electric field values and three laser parameter strengths. Dashed lines in (**d**) are depicted to visualize the changes with the electric and laser fields. The geometric parameters are fixed as H=12 nm, $R_{1}=3$ nm, $R_{2}=8$ nm, and $W_{L}=3$ nm.

Figure 6 shows an analysis of the first low-lying electron states as functions of the externally applied electric field and ILF. Figure 6a–c show the energy variation of the lowest states with quantum numbers n=0, n=1, and n=2 for three values of the laser parameters. The negative value of electric field intensity means that it points towards the negative *z*-axis. In the absence of an electric field, the energies of the different electron states decrease as the laser parameter increases. On the other hand, if the value of $\alpha_{0}$ is fixed, the energy of the states increases with the intensity of the electric field directed towards the positive *z*-direction. It decreases when the absolute value of the electric field increases if it is directed towards the negative *z*-axis. Figure 6d shows the electron probability density corresponding to the ground state with l=0 for three different values of the laser parameter and for the three extreme values of the applied electric field, i.e., F=−50, F=0, and F=50 kV/cm. In all cases it can be seen how the probability of finding the electron shifts downwards as the electric field changes from pointing toward the negative to the positive *z*-axis; the case of $\alpha_{0}=5$ nm is remarkable, where the confining effect of the ILF is so strong that the electric field effect is nearly negligible. An increase in the value of the laser parameter results in a greater penetration of the electron wavefunction into the core region, due to the reduction it causes in the CdTe barrier, as discussed in the potentials presented in Figure 2, Figure 3 and Figure 4. This justifies the greater probability of finding the electron in the core region when the electric field points towards the negative *z*-direction. The effect of the electric field on the wavefunction is more dramatically appreciated in both the absence and the ILF’s maximum value. In contrast, it is nearly neutralized for an intermediate value, indicating the competition between these fields when applied to the nanostructure. The above shows that the laser field can be adjusted to magnify or minimize the effect of the externally applied electric field.

**Figure 7:**
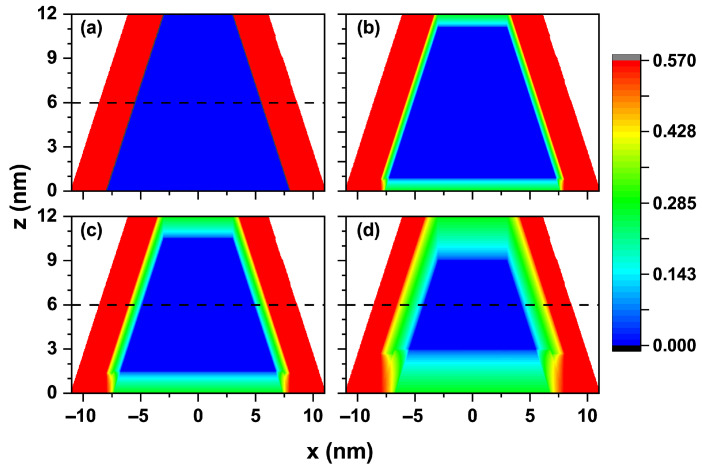
y=0-projection of the hole confinement potential in a CdTe/CdSe core/shell TCQD associated with (**a**) $\alpha_{0}=0$, (**b**) $\alpha_{0}=3$ nm, (**c**) $\alpha_{0}=5$ nm, and (**d**) $\alpha_{0}=10$ nm. The geometrical parameters of the structure are kept constant with values of $R_{1}=3$ nm, $R_{2}=8$ nm, $W_{L}=3$ nm, and H=12 nm. Additionally, F=0.

Figure 7 shows a cross-sectional view of the hole confinement potential. In this system, the hole is confined within the CdTe region, while CdSe behaves as the barrier material. Comparing Figure 7a, which corresponds to no ILF, with Figure 7b–d, where the laser parameter $\alpha_{0}$ takes values of 3 nm, 5 nm, and 10 nm, respectively, the laser shrinks the effective confinement region. The progressive evolution observed across these panels highlights how the ILF modifies the confinement potential, providing a basis for analyzing the corresponding changes in the hole energy spectrum as the laser alters the system. In contrast to the electron case (Figure 2), the effective potential of the hole shows a weaker response to increasing the laser parameter $\alpha_{0}$. Although there is a slight modulation of the potential, particularly near the base of the cone, the laser-induced effects are significantly smaller. Several physical factors can explain this difference. First, the hole has an effective mass much larger than that of the electron, which makes it less sensitive to external perturbations because of its higher inertia. Within the dressed potential model, the modulation of the confinement potential induced by the laser field is inversely proportional to the effective mass. Therefore, the impact of the laser on the hole confinement is considerably reduced. Additionally, in this type-II heterostructure, the hole is confined within the CdTe core, where the potential well is deeper (0.57 eV). This contrasts with the electron, which is located in the CdSe shell, a region with a shallower potential that is more easily altered by the laser field. As a result, the wavefunction of the hole remains more localized and centered, and the laser field does not affect either the confinement profile or the spatial distribution of the carrier. Taken together, these factors explain why the hole confinement remains with small changes with the increase in laser intensity, in contrast to the electron case, where a reduction in the confinement barrier and wavefunction redistribution are observed.

**Figure 8:**
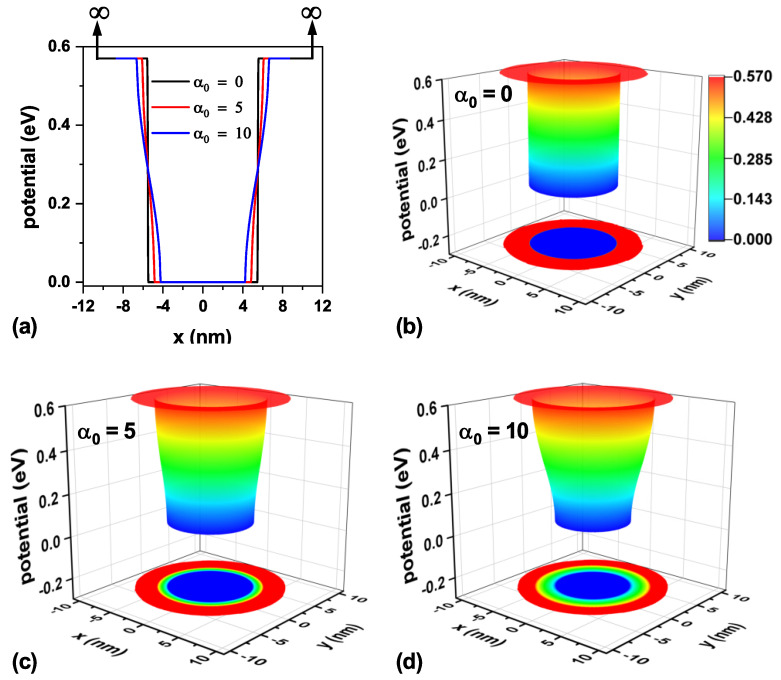
Representation of the hole confinement potential on a transverse section at half the nanostructure height, z=6 nm, for the CdTe/CdSe core/shell TCQD with geometrical parameters H=12 nm, $R_{1}=3$ nm, $R_{2}=8$ nm, and $W_{L}=3$ nm considering three different values of the laser parameter: (**a**) a 2D plot of the potential profile; 3D views and 2D projections of the confinement potential with (**b**) $\alpha_{0}=0$, (**c**) $\alpha_{0}=5$ nm, and (**d**) $\alpha_{0}=10$ nm. Additionally, F=0.

Figure 8 shows that the confinement profile mainly affects the lowest-lying states and those approaching the barrier. Because our targets are precisely these low-energy states, an ILF can be used to tune the hole states of interest. The 3D views reveal in detail how the ILF reshapes the potential; at modest laser amplitudes, the changes are subtle, so a more intense laser is required to produce confinement alterations for the hole comparable to those already obtained for the electron.

**Figure 9:**
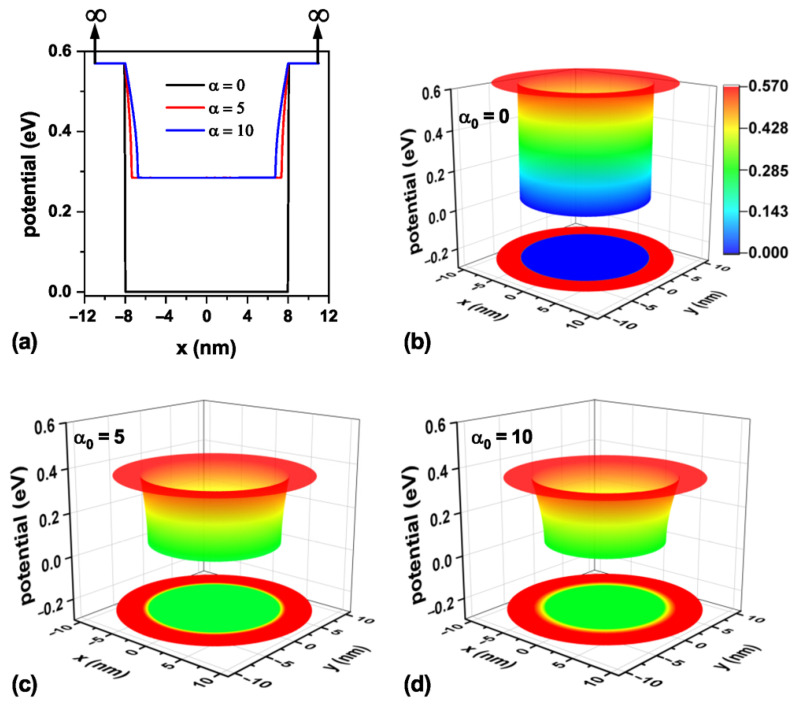
Representation of the hole confinement potential on a transverse section at the base of the nanostructure, z=0 nm, for the CdTe/CdSe core/shell TCQD with geometrical parameters H=12 nm, $R_{1}=3$ nm, $R_{2}=8$ nm, and $W_{L}=3$ nm considering three different values of the laser parameter: (**a**) a 2D plot of the potential profile; 3D views and 2D projections of the confinement potential with (**b**) $\alpha_{0}=0$, (**c**) $\alpha_{0}=5$ nm, and (**d**) $\alpha_{0}=10$ nm. Additionally, F=0.

In Figure 9, an intermediate height barrier emerges for holes, preventing any significant probability density from accumulating near the CdTe edges. Figure 9c,d confirm that the in-plane (xy) confinement is practically identical for $\alpha_{0}=5$ nm and 10 nm; the appreciable variations appear only in larger *z*, consistent with the trends discussed for Figure 8. The hole remains localized in the center of the CdTe region. The lateral confinement in the *x*–*y*-plane is almost unaffected. Even at high laser intensities, the in-plane potential remains relatively unchanged. This is due to the strong and deep potential well of the CdTe core, as well as the fact that the laser field has a limited influence over the radial direction in this region. The results confirm that the hole remains strongly confined at the base, and the laser field alone is insufficient to significantly modify its radial confinement.

**Figure 10:**
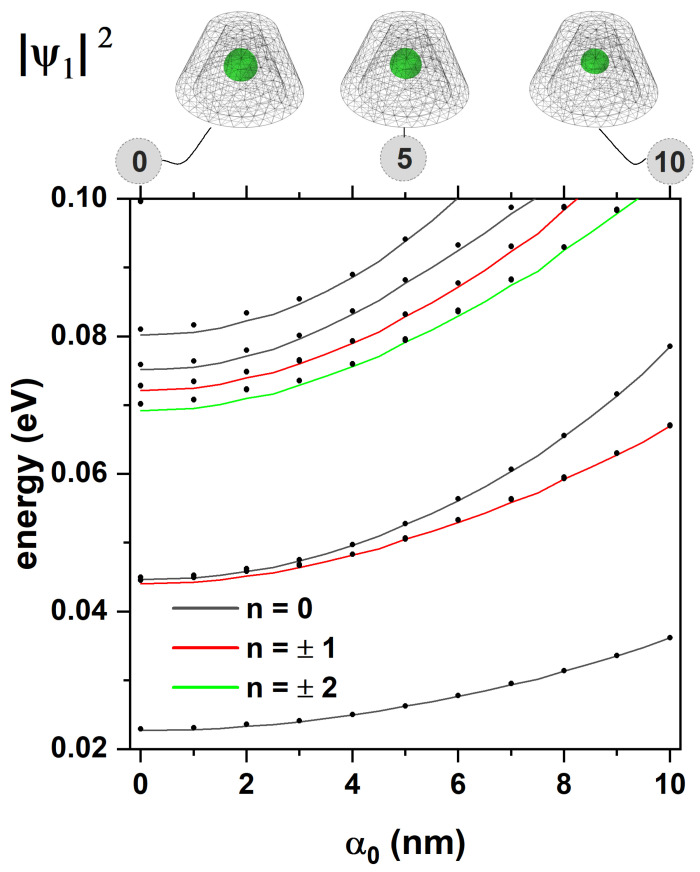
Energy spectra of the CdTe/CdSe core/shell TCQD with geometrical parameters H=12 nm, $R_{1}=3$ nm, $R_{2}=8$ nm, and $W_{L}=3$ nm as a function of the laser parameter for different values of the quantum number, *n*. Solid lines correspond to the axisymmetric 2D model, and dotted lines are obtained from a 3D model. The ground-state hole probability densities for three values of the laser parameter are also depicted. Calculations correspond to F=0.

Figure 10 shows the evolution of the hole energy levels as a function of the laser parameter $\alpha_{0}$, by using both the 2D axisymmetric model (solid lines) and the full 3D model (dotted lines). The good agreement between the two models confirms the validity of the axisymmetric approximation for describing hole states in this system. As the laser parameter increases, the energy levels of the hole also rise. In this system, the laser field does not globally reduce or increase the confinement potential. Instead, it modifies the confinement in a localized manner, particularly growing the effective confinement near the base of the truncated cone. As seen in Figure 7, Figure 8 and Figure 9, the laser-induced potential reshaping leads to stronger confinement along the *z*-axis in the lower part of the nanostructure, where the hole wavefunction is primarily located. This localized enhancement in confinement compresses the hole wavefunction along the vertical direction, shifting the energy levels upward. Meanwhile, the upper part of the barrier may experience a slight reduction in height due to the laser field. However, since the hole remains localized chiefly in the lower (CdTe) region of the structure, this has a negligible effect on the low-lying states. The probability densities plotted above Figure 10 confirm that the hole wavefunction becomes slightly more localized as $\alpha_{0}$ increases, particularly along the *z*-direction, while retaining its overall symmetry. These findings suggest that the laser field serves as a selective tuning mechanism, enhancing confinement locally rather than uniformly across the entire structure.

For holes (in Figure 10), the shift is more modest due to their larger effective mass and deeper core confinement. Although the ILF slightly narrows the confinement region, especially near the base, it does not drastically reduce the barrier height, so wavefunction compression rather than delocalization dominates. This explains the monotonic increase in energy levels with the increase in $\alpha_{0}$ and the slight vertical squeezing of the hole probability density in Figure 10. The physical cause lies in the asymmetric modification of the vertical confinement, which is selectively enhanced at the base due to the laser field’s *z*-axis polarization. These trends highlight the key role of the ILF-dressed potential in enabling state-dependent modulation of quantum confinement, offering a versatile tool for tuning both energy spectra and spatial carrier distributions in conical QDs.

**Figure 11:**
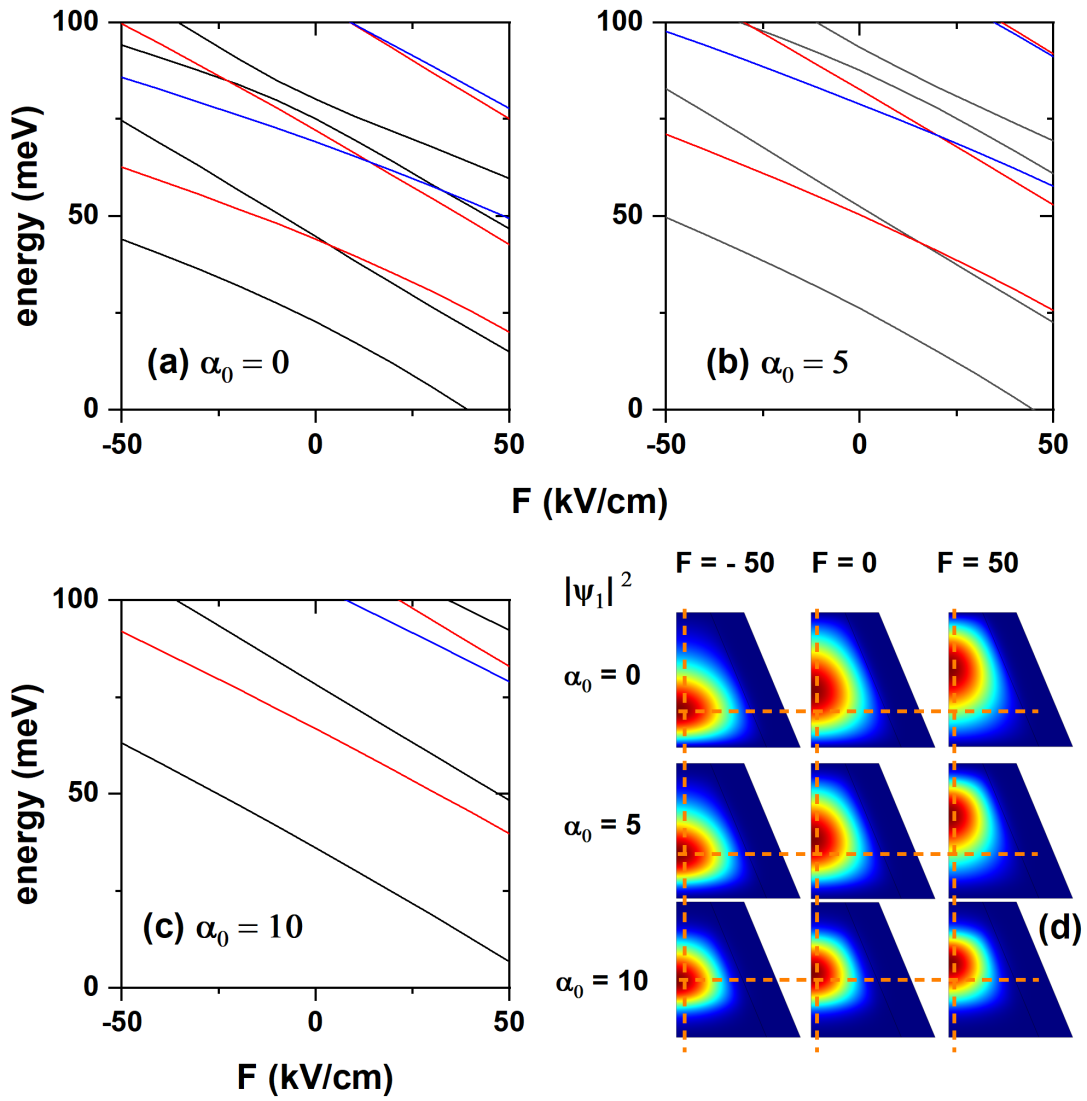
Energies of the first low-lying low states in the CdTe/CdSe core/shell TCQD are presented as functions of the externally applied electric field for three different values of the laser parameter, $\alpha_{0}=0$ (**a**), $\alpha_{0}=5$ (**b**), and $\alpha_{0}=10$ nm (**c**). In (**d**) the hole ground-state probability density for different electric field values and the three analyzed laser parameters are depicted. The dashed lines in (**d**) visualize the changes in the electric and laser fields. The geometric parameters are fixed as H=12 nm, $R_{1}=3$ nm, $R_{2}=8$ nm, and $W_{L}=3$ nm.

In Figure 11d, as one moves down the rows (i.e., as $\alpha_{0}$ increases), the laser reduces the volume of the probability density, in agreement with the dressed potential analysis. For a negative *z*-directed electric field (F=−50 kV/cm, first column), the hole, being a positive carrier, shifts in the same direction as the field; compare this with the second column, where no electric field is applied. The third column illustrates the effect of a positive *z*-directed field (F=+50 kV/cm), which pushes the hole towards the cone apex. In both field orientations, competition arises between ILF and electric field effects; for example, at $\alpha_{0}=10$ nm, the ILF dominates because the laser-induced change in effective confinement outweighs the influence of the electric field.

**Figure 12:**
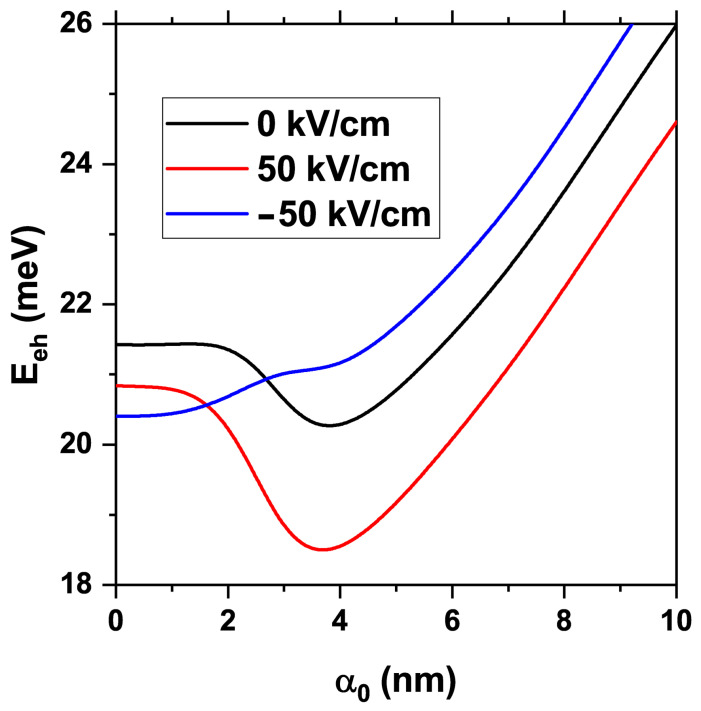
Electron–hole Coulomb energy as a function of laser parameter $\alpha_{0}$ for three fixed values of the electric field, 0, 50 kV/cm, and −50 kV/cm, in the CdTe/CdSe core/shell TCQD with the geometric parameters fixed as H=12 nm, $R_{1}=3$ nm, $R_{2}=8$ nm, and $W_{L}=3$ nm.

Figure 12 plots the electron–hole Coulomb energy versus $\alpha_{0}$ for three cases: (i) no electric field (F=0), (ii) F=+50 kV/cm, and (iii) F=−50 kV/cm. For small laser parameters, roughly $\alpha_{0}<2$ nm, the exciton energy stays constant because the individual confinement of electron and hole is not altered enough to affect their interaction. With no electric field, the Coulomb energy decreases for 2 nm $<\alpha_{0}<4$ nm: the electron is drawn to the CdSe base, while the effective region of the hole in CdTe shrinks, increasing their separation. For $\alpha_{0}>4$ nm the interaction strengthens because the electron begins to penetrate the CdTe core, triggering a transition from an indirect to a direct exciton.

When a positive electric field is applied (F=+50 kV/cm), the confinement of the carriers resembles the F=0 case, so the curve retains the same shape but shifts downward in energy: the field pulls electron and hole in opposite *z*-directions, increasing their separation. By contrast, for F=−50 kV/cm, the Coulomb energy rises monotonically—even within the 2–4 nm interval, where it previously fell. A negative *z*-directed field squeezes the hole density toward the cone base; at these $\alpha_{0}$ values, the electron still resides near the base, though in CdSe, so the carriers move closer together, enhancing their interaction. These results underscore that the combined ILF and electric field can be used to tailor excitonic properties in a highly directional and intensity-dependent way.

The results shown in Figure 12 reveal a field-controlled transition from an indirect to a direct exciton configuration, marked by a sharp increase in Coulomb interaction energy as the laser field parameter $\alpha_{0}$ exceeds 4 nm, particularly under negative electric fields. This transition is not only structural but also optically significant. In the indirect regime, where the electron resides primarily in the CdSe shell and the hole remains localized in the CdTe core, the spatial separation between carriers reduces their Coulomb attraction, resulting in weaker oscillator strength and lower radiative recombination rates. This configuration is advantageous for optoelectronic applications, where long carrier lifetimes support charge extraction. However, as $\alpha_{0}$ increases and the electron wavefunction begins to penetrate the CdTe core, the overlap between the electron and hole densities increases, transitioning the system to a direct exciton. This leads to a marked enhancement in oscillator strength, which scales roughly with the square of the electron–hole wavefunction overlap. Consequently, the radiative recombination rate is significantly increased, which is a desirable feature for quantum dot light-emitting diodes (QLEDs). Furthermore, this enhanced overlap typically results in a redshift of the optical absorption and emission peaks because the stronger Coulomb interaction reduces the total exciton energy. Such spectral tunability via external fields opens up the possibility for dynamically reconfigurable photonic devices, where the emission wavelength and efficiency can be modulated in real-time without altering the nanostructure geometry or composition. Thus, the field-driven exciton reconfiguration demonstrated here provides not only a deeper physical understanding of confinement-modulated interactions but also a technologically relevant pathway for controlling optoelectronic responses in nanoscale devices. This increase in oscillator strength, resulting from stronger electron–hole overlap, is expected to lead to higher radiative recombination rates. In practical terms, this transition from indirect to direct exciton behavior makes the structure highly promising for high-brightness QLEDs, where spatial overlap governs light emission efficiency [17,18].

While field-induced modulation of confinement can be applied broadly in nanostructured optoelectronic devices, the specific characteristics of our CdTe/CdSe TCQD structure make it particularly promising for quantum dot light-emitting diode (QLED) applications. The infinite confinement potential at the external boundary, along with the tunneling restricted to the core, ensures that charge carriers remain well-confined and spatially localized. This strong confinement suppresses carrier leakage and enables enhanced radiative recombination efficiency, key parameters for QLED performance. Moreover, the tunability offered by external fields allows dynamic control over the wavefunction overlap, enabling brightness and emission tuning without compromising structural integrity. By contrast, in photovoltaic devices, efficient charge extraction is required, which is hindered in such strongly confined systems. For example, in the work by Khan and Wang [55], optimization of all-inorganic tin-based perovskite solar cells is achieved through careful band alignment and carrier transport engineering using the SCAPS-1D simulator. Their strategy relies on material and layer tuning for efficient carrier extraction. At the same time, our system provides a field-based approach to recombination tuning, which is more relevant to emissive rather than absorptive device architectures.

To enhance the relevance of our theoretical findings, we refer to key experimental studies reporting field-tunable photoluminescence (PL) behavior in CdTe/CdSe type-II systems. Ca et al. [56] reported that CdTe/CdTeSe/CdSe core/alloyed/shell QDs exhibit redshifted PL and extended carrier lifetimes, reflecting the spatial separation of electrons and holes, a hallmark of type-II alignment. Similarly, Zhao et al. [57] observed distinct optical absorption features and PL behavior in CdTe/CdSe/CdTe nanorods, consistent with field-sensitive recombination and confinement effects. In addition, Chan et al. [7] and Rojas Valencia et al. [58] demonstrated that CdTe/CdSe core/shell QDs synthesized in noncoordinating solvents showed strong and tunable emissions in the visible range, from 570 to 610 nm, depending on the shell thickness and excitation energy. These experimental results align with our predictions on electron–hole separation, exciton behavior, and the impact of laser and electric fields on energy levels and recombination. Therefore, our model provides a theoretical foundation for designing and modulating type-II nanostructures for applications in tunable photonic and optoelectronic devices.

## 4. Conclusions

In conclusion, this work demonstrates the significant impact of combining an external electric field and an intense nonresonant laser field (ILF) on the electronic properties of a QD with a truncated cone geometry (CdTe/CdSe). The modeling, based on the effective mass approximation and finite element method, reveals that the laser field can modify the properties of the confinement in a manner comparable to geometric alterations. In particular, the laser field can transform the original ring-like QD geometry into a dot-like QD, achieved through the reduction in the potential barrier and the widening of the well at the cone base. This effect provides a valuable tool for simulating structural irregularities and changes before experimentally synthesizing the nanodevice.

Furthermore, this study highlights the role of laser intensity as a tool to control quantum confinement. At intermediate laser intensities (around $\alpha_{0}=3$ nm), a maximum in the ground-state energy is observed, suggesting a delicate balance between laser-induced effects and confinement properties. The external electric field also plays a crucial role, with its influence being amplified or attenuated depending on the laser parameters. In the presence of an intermediate laser field, the effect of the electric field becomes nearly negligible, revealing the competition between these two external fields.

The analysis of the evolution of confinement and energy spectra for both electrons and holes confirms that the interaction between the laser and electric field enables the manipulation of carrier localization, affecting their respective states. For excitons, the combined effect of these fields facilitates the tuning of the Coulomb interaction energy, allowing even a transition from an indirect to a direct exciton. This presents a promising approach for designing optoelectronic devices, as the manipulation of quantum confinement and excitonic properties opens new possibilities for the development of advanced semiconductor nanodevices.

## Data Availability

No new data were created or analyzed in this study.
